# Pseudohypoparathyroidism type 1B mimicking gitelman syndrome: diagnostic pitfalls and molecular insights

**DOI:** 10.3389/fgene.2025.1638472

**Published:** 2025-08-14

**Authors:** Yiming Zhao, Lijun Mou, Oumayma Akaaboune, Jiudan Zhang

**Affiliations:** ^1^ Department of Endocrinology and Metabolism, The Second Affiliated Hospital Zhejiang University School of Medicine, Hangzhou, China; ^2^ Department of Nephrology, The Second Affiliated Hospital Zhejiang University School of Medicine, Hangzhou, China; ^3^ Zhejiang University School of Medicine, Hangzhou, China

**Keywords:** pseudohypoparathyroidism type 1B, hypokalemia, hypocalcemia, gitelman syndrome, methylation analysis

## Abstract

**Objective:**

Pseudohypoparathyroidism type 1B (PHP1B), caused by abnormal methylation of the *GNAS* gene leading to parathyroid hormone (PTH) resistance, lacks Albright hereditary osteodystrophy features and is often misdiagnosed. PHP1B and Gitelman syndrome (GS) share overlapping features, including hypokalemia, hypocalcemia, hypomagnesemia, and metabolic alkalosis, posing challenges in clinical differentiation. This study aimed to explore the clinical characteristics, diagnostic strategies, and therapeutic responses of PHP1B presenting with hypokalemia and to explicitly address the diagnostic challenge of differentiating it from GS.

**Methods:**

Retrospective analysis of five patients initially misdiagnosed with GS due to hypokalemia but ultimately confirmed as PHP1B were collected. Whole-exome sequencing (WES) was used to exclude mutations in genes associated with renal tubular diseases, and methylation-sensitive multiplex ligation-dependent probe amplification (MS-MLPA) was employed to assess *GNAS* methylation status.

**Results:**

Patients (median age 48 years; 60% female) had prolonged diagnostic delays (median 11 years). Universal clinical manifestations included muscle cramps and weakness. Biochemical profiling revealed hypokalemia (mean potassium 3.14 mmol/L), hypocalcemia (mean calcium 1.55 mmol/L), and elevated PTH (mean 422.1 pg/mL). All patients exhibited intracranial calcifications, predominantly in the basal ganglia. Genetic testing excluded Bartter/Gitelman syndromes, while MS-MLPA identified multi-differentially methylated region defects (NESP hypermethylation with AS1/XL/A/B hypomethylation) in four patients and isolated A/B hypomethylation with heterozygous *STX16* deletion in case 5. Electrolyte levels improved with calcium, calcitriol, and potassium supplementation, though two patients required long-term potassium maintenance.

**Conclusion:**

PHP1B can present with nonspecific hypokalemia, mimicking GS. Definitive diagnosis requires combined WES and methylation analysis, particularly in WES-negative cases with PTH resistance and intracranial calcifications. Therapeutic focus should prioritize calcium/calcitriol over potassium supplementation, with epigenetic heterogeneity guiding long-term management.

## 1 Introduction

Pseudohypoparathyroidism (PHP) is a rare disorder caused by defects in the *GNAS* gene, which encodes the alpha subunit of the stimulatory G protein (Gsα) (Jüppner, 2021). Mutations or epigenetic alterations in this gene impair Gsα activity, leading to disrupted activation of Gsα-dependent signaling pathways and a diminished intracellular response to parathyroid hormone (PTH) (Pereda et al., 2021). This resistance to PTH results in hypocalcemia, hyperphosphatemia, and secondary hyperparathyroidism. In some cases, PHP is associated with Albright hereditary osteodystrophy (AHO), a distinctive phenotype characterized by round face, brachydactyly, short stature, and ectopic ossifications (Jiang et al., 2023). Resistance to multiple hormones that signal through G protein–coupled receptors, such as thyroid-stimulating hormone (TSH) and gonadotropins, may also occur in certain subtypes of PHP. PHP is classified into type 1 (PHP1) and type 2 (PHP2), with PHP1 further subdivided into PHP1A, PHP1B, and PHP1C based on clinical features and underlying molecular mechanisms (Jüppner, 2021; Mantovani et al., 2018). PHP1A is caused by heterozygous inactivating mutations in the maternally derived *GNAS* allele encoding Gsα. It is characterized by multihormone resistance (PTH, TSH, gonadotropins, etc.) and the presence of AHO features (Jiang et al., 2023). PHP1C is clinically similar to PHP1A (multihormone resistance and AHO) but exhibits normal Gsα activity in some assays, often associated with specific *GNAS* mutations affecting receptor coupling (Thiele et al., 2011). In contrast, patients with PHP1B typically exhibit isolated or predominant PTH resistance with an absence of AHO presentations and are caused by epigenetic alterations (aberrant methylation) at the *GNAS* locus (Urakawa et al., 2023), leading to delayed diagnosis and treatment.

Gitelman syndrome (GS) is an autosomal recessive salt-wasting tubulopathy caused by biallelic inactivating mutations in the *SLC12A3* gene, which encodes the thiazide-sensitive sodium-chloride cotransporter in the distal convoluted tubule. The resulting electrolyte disturbances include hypokalemic metabolic alkalosis, hypomagnesemia, and elevated plasma renin activity. GS presents with a broad spectrum of clinical symptoms and carries risks of serious complications such as cardiac arrhythmias and rhabdomyolysis (Blanchard et al., 2017). In some atypical cases, GS may also be associated with hypocalcemia and secondary elevations in PTH levels, further complicating differential diagnosis (Verploegen et al., 2022).

Here, we report five unrelated patients with PHP1B who were initially misdiagnosed with GS due to overlapping clinical features—namely, hypokalemia, hypocalcemia, and elevated renin activity. Subsequent genetic and epigenetic investigations led to a revised diagnosis of PHP1B. This study delineates the clinical and biochemical features of PHP1B presenting with hypokalemia, evaluates the diagnostic pitfalls underlying its misdiagnosis with GS, and assess therapeutic responses.

## 2 Subjects and methods

### 2.1 Study design and ethical considerations

This retrospective cohort study was approved by the Institutional Review Board of the Second Affiliated Hospital of Zhejiang University School of Medicine (2022-0921). All procedures followed the Declaration of Helsinki. Written informed consent was obtained from all participants.

### 2.2 Subjects and data collection

Five patients initially misdiagnosed with Gitelman syndrome or hypokalemia of unknown origin were enrolled based on: (1) biochemical confirmation of hypokalemia, hypocalcemia, and elevated PTH; (2) absence of AHO features; (3) exclusion of Gitelman/Bartter mutations. Clinical data were extracted from electronic medical records, including demographics, symptom duration, family history, therapeutic interventions, serial electrolyte measurements (calcium, potassium, phosphorus, magnesium), and hormonal profiles (PTH, renin, aldosterone, TSH). Neuroimaging reports were reviewed for intracranial calcifications.

### 2.3 Biochemical analysis

Serum electrolytes were quantified using ion-selective electrodes (Beckman Coulter AU Series; reference ranges: potassium 3.5–5.3 mmol/L, calcium 2.11–2.52 mmol/L, magnesium 0.75–1.02 mmol/L, phosphorus 0.85–1.51 mmol/L). PTH was measured by electrochemiluminescence immunoassay (Roche Cobas e801; normal 15–65 pg/mL). Total 25-hydroxyvitamin D (25OHD) was assessed via electrochemiluminescence immunoassay (Roche Cobas e801; deficiency <20 ng/mL). Plasma renin and aldosterone were determined by chemiluminescence immunoassay (DiaSorin; upright renin 4.4–46.1 uIU/mL, aldosterone 30–353 pg/mL). TSH was measured by chemiluminescence microparticle immunoassay (Abbott ARCHITECT i2000SR; normal 0.35–4.94 mIU/L).

### 2.4 Genetic and epigenetic analysis

Whole-exome sequencing (Illumina NovaSeq 6000, 150-bp paired-end) screened pathogenic variants in genes associated with renal tubular disorders: *SLC12A3* (Gitelman syndrome); *SLC12A1*, *CLCNKB*, *BSND*, *KCNJ1*, *MAGED2* (Bartter syndrome); *CASR*, *CLCNKA*, *KCNJ10* (other hypokalemia-related genes). This comprehensive screening strategy was essential to exclude all major renal salt-wasting disorders. Methylation status at *GNAS* differentially methylated regions (DMRs) was assessed using the SALSA multiplex ligation-dependent probe amplification (MS-MLPA) ME031-B1 probemix (MRC Holland). Briefly, genomic DNA (200 ng) was denatured, followed by probe hybridization. Subsequently, samples were divided into two tubes, one without HhaI digestion, undergoing ligation only, and the other undergoing simultaneously ligated and digested by *Hha*I. Both reactions were then subjected to PCR amplification. Fragment analysis used capillary electrophoresis (ABI 3500xl). Methylation ratios were calculated in Coffalyser.Net v.220621 by relative probe peaks between the undigested sample and the digested sample for each probe.

## 3 Results

### 3.1 Clinical presentation

Five patients (3 females and 2 males; median age 48 years) with pseudohypoparathyroidism type 1B (clinical characteristics summarized in Table 1) demonstrated prolonged diagnostic delay (median 11 years, range 8–60). Case 1, a 51-year-old male, presented with progressive lower limb weakness and muscle spasms persisting for 8 years, previously misdiagnosed as Gitelman syndrome and treated with potassium-sparing diuretics and magnesium supplementation without symptomatic relief. Case 2, a 32-year-old male, reported 12 years of episodic limb stiffness and muscle weakness, having received intermittent calcium and potassium supplementation at local hospitals. Case 3, a 23-year-old female, was incidentally diagnosed through routine blood tests showing asymptomatic hypokalemia and hypocalcemia, with no prior therapeutic interventions. Case 4, a 60-year-old female presented with short stature since childhood; her laboratory profile demonstrated persistent hypokalemia and hypocalcemia. Case 5, a 48-year-old female, experienced recurrent muscle weakness for 10 years, initially responsive to potassium chloride supplementation but relapsing upon discontinuation; notably, her younger brother reported similar undiagnosed neuromuscular symptoms. Critically, no patient exhibited brachydactyly, subcutaneous ossifications, or other AHO features.

**TABLE 1:** Clinical characteristics of 10 PHP1B patients with hypokalemia.

Patient No.	1	2	3	4	5	6 (Zhang et al., 2022)	7 (Yang et al., 2022)	8 (Lecumberri et al., 2022)	9 (Lecumberri et al., 2022)	10 (Huang et al., 2022)
Age at Diagnosis (Year)	51	32	23	60	48	26	13	34	23	27
Gender	Male	Male	Female	Female	Female	Female	Female	Male	Female	Female
Symptoms at Diagnosis	Cramping, Weakness	Muscle Spasm, Weakness	No Symptom	Short Stature	Weakness	Tetany, Weakness	Tetany, Seizures	Seizure	Seizure	Tetany
Duration (Year)	8	12	NA	Nearly 60	10	10	5	11	6	1
Family History	No	No	No	No	Younger Brother	Mother (Probable)	No	No	No	No
AHO Features	No	No	No	No	No	No	No	No	No	No
Short Stature	No	No	No	Yes	No	Yes	Yes	Yes	No	No
Obesity	No	No	No	No	No	Yes	No	No	No	No

AHO, albright hereditary osteodystrophy; NA, not available.

### 3.2 Laboratory and imaging findings

**FIGURE 1:**
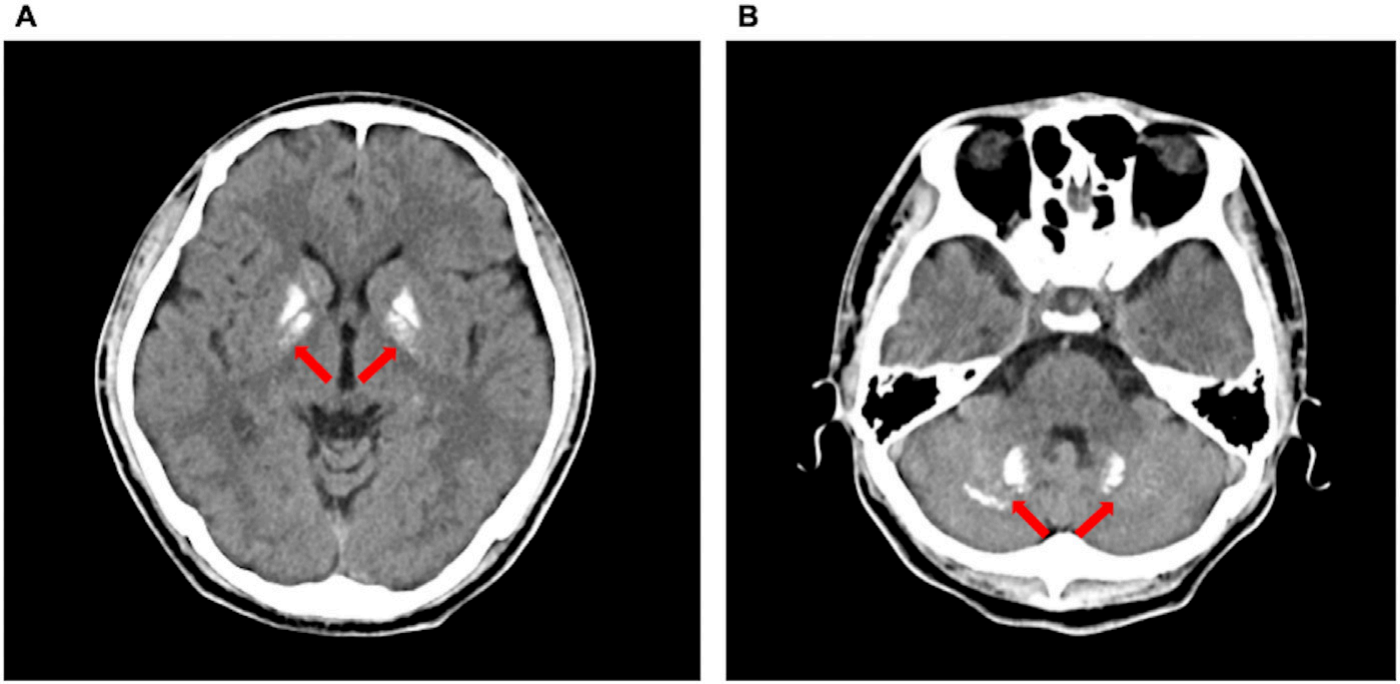
Non-contrast computed tomography showed intracranial calcifications (arrows) in Case 2, including bilateral basal ganglia **(A)** and bilateral dentate nuclei of the cerebellum **(B)**.

All patients exhibited the characteristic biochemical profile of PHP1B (Table 2): severe hypocalcemia (mean serum calcium: 1.55 mmol/L), hypokalemia (mean serum potassium: 3.14 mmol/L), and elevated PTH (mean PTH: 422.1 pg/mL). Additional abnormalities included hypomagnesemia in three patients (mean serum magnesium: 0.74 mmol/L), vitamin D deficiency in two (mean 25OHD: 27.6 ng/mL), hyperphosphatemia in two (mean phosphorus 1.41 mmol/L), and universal elevated plasma renin activity. Neuroimaging via non-contrast computed tomography revealed intracranial calcifications in all cases (Figure 1; Supplementary Figure S1). Dual-energy X-ray absorptiometry (DXA) confirmed osteoporosis in Cases 1 and 3 (Supplementary Figure S2).

**TABLE 2:** Laboratory and imaging findings of 10 PHP1B patients at diagnosis.

Patient No.	1	2	3	4	5	6 (Zhang et al., 2022)	7 (Yang et al., 2022)	8 (Lecumberri et al., 2022)	9 (Lecumberri et al., 2022)	10 (Huang et al., 2022)
Calcium (mmol/L)	1.63	1.19	1.48	1.79	1.67	1.35	1.65	1.58	1.55	1.77
PTH (pg/mL)	764.2	342.1	591.9	97.49	315	201.4	322.2	461	175	422.2
Potassium (mmol/L)	3.11	3.15	3.04	3.27	3.14	2.9	2.43	NA	NA	3.13
Hyperphosphatemia	No	No	No	NA	Yes	Yes	Yes	Yes	Yes	Yes
Hypomagnesemia	Yes	Yes	Yes	No	No	No	No	NA	NA	No
Low 25OHD	No	Yes	Yes	No	No	Yes	Yes	NA	NA	No
High Renin Activity	Yes	Yes	Yes	Yes	Yes	Yes	Yes	NA	NA	Yes
High TSH	No	No	No	Na	No	No	Yes	Yes	Yes	No
Intracranial Calcifications (Location)	Left Centrum Semiovale	Bilateral Dentate Nuclei of the Cerebellum, Basal Ganglia	Basal Ganglia	Bilateral Centra Semiovalia, Basal Ganglia, Cerebellum	Cerebellum, Basal Ganglia, Right Frontal Lobe	Bilateral Frontal Lobe and Basal Ganglia	Basal Ganglia	Clivus, Cerebral Parenchyma, Cerebellum, Basal Ganglia	Cerebral Parenchyma, Cerebellum, Basal Ganglia	Basal Ganglia
Osteoporosis	Yes	No	Yes	NA	NA	NA	NA	NA	NA	NA

PTH, parathyroid hormone; 25OHD, 25-hydroxyvitamin D; TSH, thyroid-stimulating hormone; NA, not available.

Reference ranges: calcium, 2.11–2.52 mmol/L; PTH, 15–65 pg/mL; potassium, 3.5–5.3 mmol/L; hyperphosphatemia, phosphorus >1.51 mmol/L; hypomagnesemia, magnesium <0.75 mmol/L; Low 25OHD, <20 ng/mL). High Renin Activity, upright renin >46.1 uIU/mL; High TSH, >4.94 mIU/L; osteoporosis, Z-score ≤ −2.0 in premenopausal women and men <50 years, T-score ≤ −2.5 in postmenopausal women and men ≥50 years at lumbar spine/femoral neck.

### 3.3 Genetic and epigenetic analysis results

**FIGURE 2:**
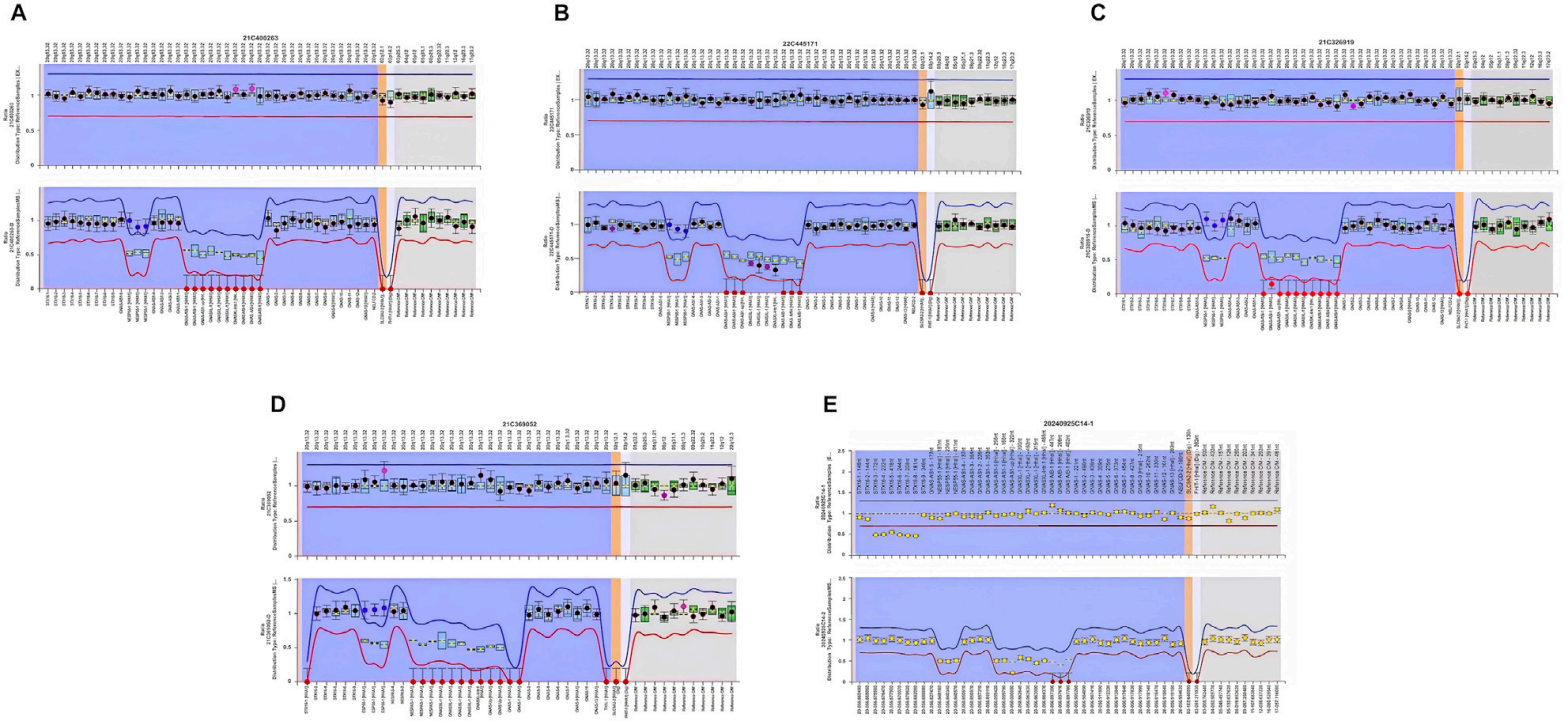
Methylation analysis of the *GNAS* locus in five patients with PHP1B. Methylation status of the differentially methylated regions (DMRs) at the *GNAS* locus was assessed in peripheral blood DNA using methylation-specific multiplex ligation-dependent probe amplification (MS-MLPA). In the figure, red dots indicate loss of methylation (hypomethylation) and purple dots indicate gain of methylation (hypermethylation). All five patients demonstrated methylation abnormalities at one or more *GNAS* DMRs: **(A)** Case 1 exhibited loss of methylation at the AS1, XL, and A/B DMRs, along with gain of methylation at the NESP DMR. **(B)** Case 2 showed loss of methylation at the AS1 and A/B DMRs, and gain of methylation at the NESP DMR. **(C)** Case 3 demonstrated loss of methylation at the AS1, XL, and A/B DMRs, with gain of methylation at the NESP DMR. **(D)** Case 4 revealed loss of methylation at the AS1 and A/B DMRs, and gain of methylation at the NESP DMR. **(E)** Case 5 had an *STX16* deletion, associated with isolated loss of methylation at the A/B DMR, consistent with autosomal dominant PHP1B.

Whole-exome sequencing (WES) excluded pathogenic variants in renal tubulopathy genes including *SLC12A3* (Gitelman syndrome), *SLC12A1*, *CLCNKB*, *BSND*, *KCNJ1*, *MAGED2* (Bartter syndrome), and *CASR*, *CLCNKA*, *KCNJ10* (hypokalemia-associated genes). Case 5 harbored a heterozygous 3.0-kb deletion at chr20:57242542-57248800 (GRCh37/hg19) spanning exons 3–8 of the *STX16* gene (NM_001001433.3), this deletion impacts a cis-acting control element essential for *GNAS* exon A/B methylation, consistent with autosomal dominant PHP1B. MS-MLPA revealed universal loss of methylation at the *GNAS* A/B-DMR (Table 3; Figure 2). Four patients demonstrated NESP-DMR hypermethylation and AS1-DMR hypomethylation, while two exhibited XL-DMR hypomethylation. Case 5 showed isolated A/B-DMR abnormality with normal methylation at other loci.

**TABLE 3:** Genetic features of the 10 patients diagnosed with PHP1B and hypokalemia.

Patient No.	1	2	3	4	5	6 (Zhang et al., 2022)	7 (Yang et al., 2022)	8 (Lecumberri et al., 2022)	9 (Lecumberri et al., 2022)	10 (Huang et al., 2022)
*STX16* microdeletion	No	No	No	No	Yes	No	No	No	No	No
Methylation pattern of the DMRs on the *GNAS* locus										
*NESP*-DMR	H	H	H	H	N	H	H	NA	NA	H
*AS1-*DMR	L	L	L	L	N	L	L	NA	NA	N
*XL-*DMR	L	N	L	N	N	L	L	L	L	N
*A/B-*DMR	L	L	L	L	L	L	L	L	L	L

DMR, differentially methylated region; N, normal; L, low; H, high.

### 3.4 Treatment response and outcomes

Prior misdiagnosis as GS prompted targeted regimens including potassium chloride (2–4 g/day in all patients), calcium carbonate (600–1,200 mg/day in all patients), spironolactone (40 mg/day in Cases 1 and Case 3), and magnesium supplementation (in Cases 1 and Case 3). These achieved partial potassium normalization in three patients (Cases 1, 2, and 5) but failed to correct hypocalcemia or normalize PTH, with symptom recurrence in all three patients upon dose reduction. Following PHP1B diagnosis, therapy was optimized to oral calcium carbonate (1,200-1,800 mg elemental calcium daily), calcitriol (0.5–0.75 µg daily), and potassium chloride (1–2g/day). Post-intervention evaluation at 3 months revealed significant symptomatic improvement, along with normalization of serum calcium (post-treatment mean 2.20 mmol/L) and potassium (post-treatment mean 3.84 mmol/L). PTH levels significantly decreased post-treatment (mean 324.1 pg/mL). In some patients, potassium supplementation was successfully discontinued following normalization of serum calcium levels; however, two patients (Case 1 and Case 3) experienced recurrent hypokalemia after withdrawal of potassium therapy.

## 4 Discussion

### 4.1 Molecular pathogenesis and clinical spectrum

Epigenetic alterations involving the *GNAS* locus, particularly hypomethylation at the exon A/B DMR, constitute the molecular hallmark of PHP1B, leading to tissue-specific paternal imprinting and silencing of Gsα expression in proximal renal tubules (Jüppner, 2021; Mantovani et al., 2018). These methylation defects result in PTH resistance, clinically manifesting as hypocalcemia, which in turn contribute to symptoms such as muscle cramps and tetany (Jüppner, 2021; Mantovani et al., 2018; Bove-Fenderson and Mannstadt, 2018). Our cohort demonstrates that PHP1B can present with significant hypokalemia and hypomagnesemia—features classically associated with GS. Analysis integrating five literature cases (Zhang et al., 2022; Yang et al., 2022; Lecumberri et al., 2022; Huang et al., 2022) reveals consistent patterns: female predominance (70%), diagnostic delay (median 10 years), and absence of AHO features. Biochemical hallmarks include hypokalemia (mean 3.02 mmol/L), hypocalcemia (mean 1.57 mmol/L), elevated PTH (mean 369.2 pg/mL), hyperphosphatemia (60%), hypomagnesemia (30%), and vitamin D deficiency (40%) (Table1 and 2). These overlapping electrolyte imbalances, combined with the frequent presence of secondary hyperaldosteronism, closely mimicked the phenotype of GS and significantly complicated the diagnostic workup. Notably, intracranial calcifications were detected in all patients at the time of diagnosis. Given the 100% prevalence of this finding—including in asymptomatic patients—we recommend routine non-contrast brain computed tomography for all PHP1B patients at diagnosis, irrespective of neurological symptoms. Several patients developed seizures or transient episodes of loss of consciousness requiring ongoing antiepileptic management (Khan and Khan, 2025), while electrolyte disturbances contributed to cardiac arrhythmias, including palpitations and electrocardiographic abnormalities. These complications pose significant risks for life-threatening events. Critically, delayed diagnosis significantly increased the risk of both acute and chronic sequelae, highlighting the need for early recognition and comprehensive evaluation in patients presenting with overlapping features of renal and endocrine dysfunction.

### 4.2 Diagnostic challenges and refinements

The clinical overlap between PHP1B and other renal and endocrine disorders poses significant diagnostic challenges. To aid in clinical classification, the European Network for the Study of Pseudohypoparathyroidism (EuroPHP Network) introduced the inactivating PTH/PTHrP signaling disorder (iPPSD) system, a molecularly based taxonomy using numbered subtypes to delineate genotypic and phenotypic features (Pereda et al., 2021). According to this classification, iPPSD3 corresponds to *GNAS* methylation defects and primarily manifests as isolated PTH resistance, consistent with prior descriptions of PHP1B (Elli et al., 2016). Despite its utility in categorizing known entities, the iPPSD system remains limited in facilitating early diagnosis, particularly in atypical presentations with overlapping phenotypes.

Through our case series and comprehensive literature review, we identified several nonspecific clinical and laboratory findings that underscore the need for a refined diagnostic algorithm. While WES is a powerful tool for detecting coding region mutations, it is insufficient for identifying epigenetic alterations characteristic of PHP1B. Given the epigenetic etiology of PHP1B, we advocate concurrent WES and targeted *GNAS* methylation analysis in patients meeting any of the following criteria: biochemical triad of hypokalemia with concomitant hypocalcemia and elevated PTH; intracranial calcifications on neuroimaging; and suboptimal or transient responses to targeted electrolyte supplementation.

### 4.3 Pathophysiological mechanisms of hypokalemia

The pathophysiological basis of hypokalemia in PHP1B remains incompletely understood but is likely multifactorial. Proposed mechanisms include renal resistance to PTH, leading to reduced production or impaired signaling of cyclic adenosine monophosphate (Jüppner, 2021). This disruption may inhibit potassium channel function in the apical membrane of the thick ascending limb of Henle’s loop, impairing potassium recycling. Consequently, diminished intraluminal potassium concentration inhibits the Na^+^-K^+^-2Cl^-^ cotransporter, exacerbating sodium loss. In response, increased sodium reabsorption in the distal tubule, via exchange for potassium and hydrogen ions, promotes urinary potassium wasting and contributes to hypokalemia (Weinstein, 2010). Chronic hypocalcemia may further impair potassium reabsorption by inducing degenerative changes in renal tubular epithelial cells or stimulating sympathetic activity, which shifts potassium intracellularly. Vitamin D_3_ deficiency, commonly observed in PHP1B, may exacerbate hypokalemia through activation of the renin–angiotensin system (Li et al., 2002). Secondary hyperaldosteronism, observed in our cohort, compounded the diagnostic complexity, particularly in differentiating PHP1B from GS. One previously reported case described coexistent PHP1B and GS presenting as refractory hypokalemia, requiring a combination of calcitriol, calcium, and potassium supplementation (Zhang et al., 2024). In contrast, WES in our patients revealed no mutations in genes associated with Bartter or Gitelman syndromes, reinforcing the importance of molecular and epigenetic testing in the differential workup. Notably, the *GNAS*-hypokalemia association currently derives from physiological inference rather than direct molecular evidence–a gap necessitating targeted epigenotype-phenotype studies.

The presence and severity of electrolyte imbalances in PHP1B may correlate with specific genetic and epigenetic signatures. PHP1B is typically associated with hypomethylation of the A/B DMR; however, methylation abnormalities in other DMRs—such as NESP, AS1, and XL—vary depending on the underlying molecular lesion. In Urakawa’s study of 84 PHP1B patients, broader methylation disturbances were associated with more severe phenotypes (Urakawa et al., 2023). Subgroup analyses demonstrated that patients exhibiting hypermethylation of the NESP-DMR along with hypomethylation of the AS1-, XL-, and A/B-DMRs had the earliest median age of onset and the most profound hypocalcemia, suggesting that the extent of methylation disruption may influence disease severity and progression (Urakawa et al., 2023; Bove-Fenderson and Mannstadt, 2018). Notably, Case 5 harbored a heterozygous *STX16* deletion—a recognized cause of autosomal dominant PHP1B (Pereda et al., 2021; Yang et al., 2022), manifesting comparatively milder electrolyte disturbances, normal renin activity, and absence of severe neurological manifestations. This attenuated phenotype correlated molecularly with a restricted epigenetic defect: isolated loss of methylation at the A/B DMR. In contrast, broader methylation disruptions involving NESP hypermethylation with AS1/XL/A/B hypomethylation predominated in other patients (Cases 1–4) and literature cohorts. Critically, 81.8% of PHP1B patients with hypokalemia exhibit these multi-DMR defects, which associate with more profound hypocalcemia and higher rates of neurological complications (Urakawa et al., 2023; Iwasaki et al., 2025). Recent mechanistic studies suggest that maternal deletions involving exon H/AS lead to AS1-DMR hypomethylation, bidirectional expression of the *GNAS*-AS1 antisense transcript, and subsequent NESP hypermethylation, pointing to complex transcriptional regulation across the *GNAS* locus (Iwasaki et al., 2025). The potential interplay between bidirectional transcription and epigenetic regulation in the pathogenesis of hypokalemia in PHP1B warrants further investigation.

### 4.4 Therapeutic management and future directions

Given the absence of curative therapies, long-term management remains supportive, typically involving calcium, potassium, and active vitamin D supplementation alongside regular biochemical monitoring. Crucially, therapeutic strategies diverge fundamentally between PHP1B and GS. In PHP1B, prompt correction of hypocalcemia with calcium and active vitamin D (calcitriol) supplementation is paramount. This intervention typically resolves concomitant hypokalemia in the majority of patients, thereby obviating the need for ongoing potassium replacement. This stands in contrast to GS management, which mandates lifelong potassium/magnesium supplementation, with calcitriol has no defined role (Blanchard et al., 2017). Notably, patients requiring sustained potassium therapy (Cases 1 and 3) concurrently exhibited hypomagnesemia and osteoporosis, along with the most extensive DMR involvement. This clinical-epigenetic correlation indicates that DMR abnormality breadth may serve as a predictor of disease severity. Consequently, comprehensive baseline assessment—including bone mineral density evaluation-is warranted. Furthermore, targeted therapeutic intensification focused on parathyroid hormone suppression should be strongly considered in patients with multi-DMR involvement.

Experimental strategies targeting methylation defects are emerging as potential disease-modifying therapies. Epigenetic agents such as DNA methyltransferase inhibitors and histone deacetylase inhibitors have demonstrated synergistic effects in preclinical studies, offering a possible future avenue for targeted therapy in PHP1B (Mu et al., 2024). Furthermore, recent advances in CRISPR–dCas9-based epigenetic editing have enabled locus-specific modulation of methylation at the *GNAS* locus. Single-cell epigenomic profiling may enhance our ability to stratify patients and design individualized correction strategies (Deshmukh et al., 2025). These innovations hold significant promise for translating molecular insights into precision therapies that may improve adherence, reduce complications, and optimize clinical outcomes in patients with PHP1B.

Study limitations include small sample size, restricted longitudinal follow-up, and the absence of functional validation for the identified epigenetic alterations. Future investigations should prioritize: functional interrogation of the epigenetic mechanisms implicated in PHP1B pathogenesis and severity; exploration of targeted epigenetic-modifying therapies and prospective validation of the proposed integrated diagnostic algorithm for hypokalemic patients. This multifaceted strategy, integrating biochemical, epigenetic, and clinical markers, establishes a framework for molecular stratification of PHP1B severity and refines precision therapeutic strategies.

## 5 Conclusion

This clinical report highlights the diagnostic complexity of PHP1B, particularly in cases presenting with overlapping electrolyte disturbances. Although hypokalemia is not traditionally considered a defining feature of PHP1B, its presence has been increasingly documented in the literature. Importantly, a negative WES result in patients exhibiting hypokalemia, hypocalcemia, and hypomagnesemia does not exclude PHP1B, especially given its epigenetic etiology. Recognizing this association is critical for timely and accurate diagnosis, as well as for the implementation of appropriate therapeutic interventions. Further research is warranted to elucidate the underlying mechanisms of hypokalemia in PHP1B—particularly those involving methylation defects at the *GNAS* locus—and to inform the development of targeted, mechanism-based treatment strategies.

## Data Availability

The original contributions presented in the study are included in the article/Supplementary Material, further inquiries can be directed to the corresponding author.

## References

[B1] Blanchard A. Bockenhauer D. Bolignano D. Calò L. A. Cosyns E. Devuyst O. (2017). Gitelman syndrome: consensus and guidance from a kidney disease: improving global outcomes (KDIGO) controversies conference. Kidney Int. 91 (1), 24–33. 10.1016/j.kint.2016.09.046 28003083

[B2] Bove-Fenderson E. Mannstadt M. (2018). Hypocalcemic disorders. Best. Pract. Res. Clin. Endocrinol. Metab. 32 (5), 639–656. 10.1016/j.beem.2018.05.006 30449546

[B3] Deshmukh M. G. Brooks V. T. Roy S. F. Milette S. Bosenberg M. Micevic G. (2025). DNA methylation in melanoma immunotherapy: mechanisms and therapeutic opportunities. Clin. Epigenetics 17 (1), 71. 10.1186/s13148-025-01865-5 40307913 PMC12044936

[B4] Elli F. M. Linglart A. Garin I. de Sanctis L. Bordogna P. Grybek V. (2016). The prevalence of GNAS deficiency-related diseases in a large cohort of patients characterized by the EuroPHP network. J. Clin. Endocrinol. Metab. 101 (10), 3657–3668. 10.1210/jc.2015-4310 27428667

[B5] Huang S. He Y. Lin X. Sun S. Zheng F. (2022). Clinical and genetic analysis of pseudohypoparathyroidism complicated by hypokalemia: a case report and review of the literature. BMC Endocr. Disord. 22 (1), 98. 10.1186/s12902-022-01011-9 35410271 PMC9004107

[B6] Iwasaki Y. Reyes M. Ryabets-Lienhard A. Gales B. Linglart A. Miller D. E. (2025). Bidirectional disruption of GNAS transcripts causes broad methylation defects in pseudohypoparathyroidism type 1B. Proc. Natl. Acad. Sci. U. S. A. 122 (16), e2423271122. 10.1073/pnas.2423271122 40249781 PMC12037034

[B7] Jiang S. Yang Y. Song A. Jiang Y. Jiang Y. Li M. (2023). Genotype-phenotype correlations in pseudohypoparathyroidism type 1a patients: a systemic review. Eur. J. Endocrinol. 189 (5), S103–s111. 10.1093/ejendo/lvad142 37837607

[B8] Jüppner H. (2021). Molecular definition of pseudohypoparathyroidism variants. J. Clin. Endocrinol. Metab. 106 (6), 1541–1552. 10.1210/clinem/dgab060 33529330 PMC8118362

[B9] Khan S. Khan A. A. (2025). Hypoparathyroidism: diagnosis, management and emerging therapies. Nat. Rev. Endocrinol. 21, 360–374. 10.1038/s41574-024-01075-8 39905273

[B10] Lecumberri S. B. Ruiz Sánchez J. G. de León Fuentes B. Álvarez Escolá C. Herranz de la Morena L. (2022). Intracranial calcifications in pseudohypoparathyroidism type 1b: report of four cases. Endocrinol. Diabetes Nutr. Engl. Ed. 69 (1), 70–72. 10.1016/j.endien.2020.09.008 35232562

[B11] Li Y. C. Kong J. Wei M. Chen Z. F. Liu S. Q. Cao L. P. (2002). 1,25-Dihydroxyvitamin D(3) is a negative endocrine regulator of the renin-angiotensin system. J. Clin. Invest 110 (2), 229–238. 10.1172/jci15219 12122115 PMC151055

[B12] Mantovani G. Bastepe M. Monk D. de Sanctis L. Thiele S. Usardi A. (2018). Diagnosis and management of pseudohypoparathyroidism and related disorders: first international consensus statement. Nat. Rev. Endocrinol. 14 (8), 476–500. 10.1038/s41574-018-0042-0 29959430 PMC6541219

[B13] Mu S. Wang W. Liu Q. Ke N. Li H. Sun F. (2024). Autoimmune disease: a view of epigenetics and therapeutic targeting. Front. Immunol. 15, 1482728. 10.3389/fimmu.2024.1482728 39606248 PMC11599216

[B14] Pereda A. Elli F. M. Thiele S. de Sanctis L. Rothenbuhler A. Hanna P. (2021). Inactivating PTH/PTHrP signaling disorders (iPPSDs): evaluation of the new classification in a multicenter large series of 544 molecularly characterized patients. Eur. J. Endocrinol. 184 (2), 311–320. 10.1530/eje-20-0625 33270042

[B15] Thiele S. de Sanctis L. Werner R. Grötzinger J. Aydin C. Jüppner H. (2011). Functional characterization of GNAS mutations found in patients with pseudohypoparathyroidism type ic defines a new subgroup of pseudohypoparathyroidism affecting selectively Gsα-receptor interaction. Hum. Mutat. 32 (6), 653–660. 10.1002/humu.21489 21488135 PMC3103608

[B16] Urakawa T. Sano S. Kawashima S. Nakamura A. Shima H. Ohta M. (2023). (Epi)genetic and clinical characteristics in 84 patients with pseudohypoparathyroidism type 1B. Eur. J. Endocrinol. 189 (6), 590–600. 10.1093/ejendo/lvad163 38039118

[B17] Verploegen M. F. A. Vargas-Poussou R. Walsh S. B. Alpay H. Amouzegar A. Ariceta G. (2022). Parathyroid hormone and phosphate homeostasis in patients with bartter and Gitelman syndrome: an international cross-sectional study. Nephrol. Dial. Transpl. 37 (12), 2474–2486. 10.1093/ndt/gfac029 PMC9681919 35137195

[B18] Weinstein A. M. (2010). A mathematical model of rat ascending henle limb. III. Tubular function. Am. J. Physiol. Ren. Physiol. 298 (3), F543–F556. 10.1152/ajprenal.00232.2009 PMC2838601 19923413

[B19] Yang W. J. Zhang Q. Jin P. (2022). A case of sporadic pseudohypoparathyroidism type 1B presented with hypokalemia. Horm. Metab. Res. 54 (1), 50–51. 10.1055/a-1528-7471 34327681

[B20] Zhang Y. Song X. Zhang W. Qi T. Sun W. Zhou X. (2022). Sporadic pseudohypoparathyroidism type 1B due to methylation abnormality combined with hypokalemia: a case report and review. Ann. Endocrinol. Paris. 83 (6), 472–474. 10.1016/j.ando.2022.09.022 36371350

[B21] Zhang X. Bi X. Wu Y. Xu P. (2024). Interpreting epigenetic causes of recurrent hypokalemia and seizures: gitelman syndrome co-exist with pseudohypoparathyroidism type 1B. Nephrol. Carlt. 29 (5), 300–304. 10.1111/nep.14270 38233937

