# Supplementary figures and images for: Pseudohypoparathyroidism type 1B mimicking gitelman syndrome: diagnostic pitfalls and molecular insights
Source: Front Genet. 2025 Aug 14;16:1638472. doi: 10.3389/fgene.2025.1638472 (PMC12390988; doi:10.3389/fgene.2025.1638472)

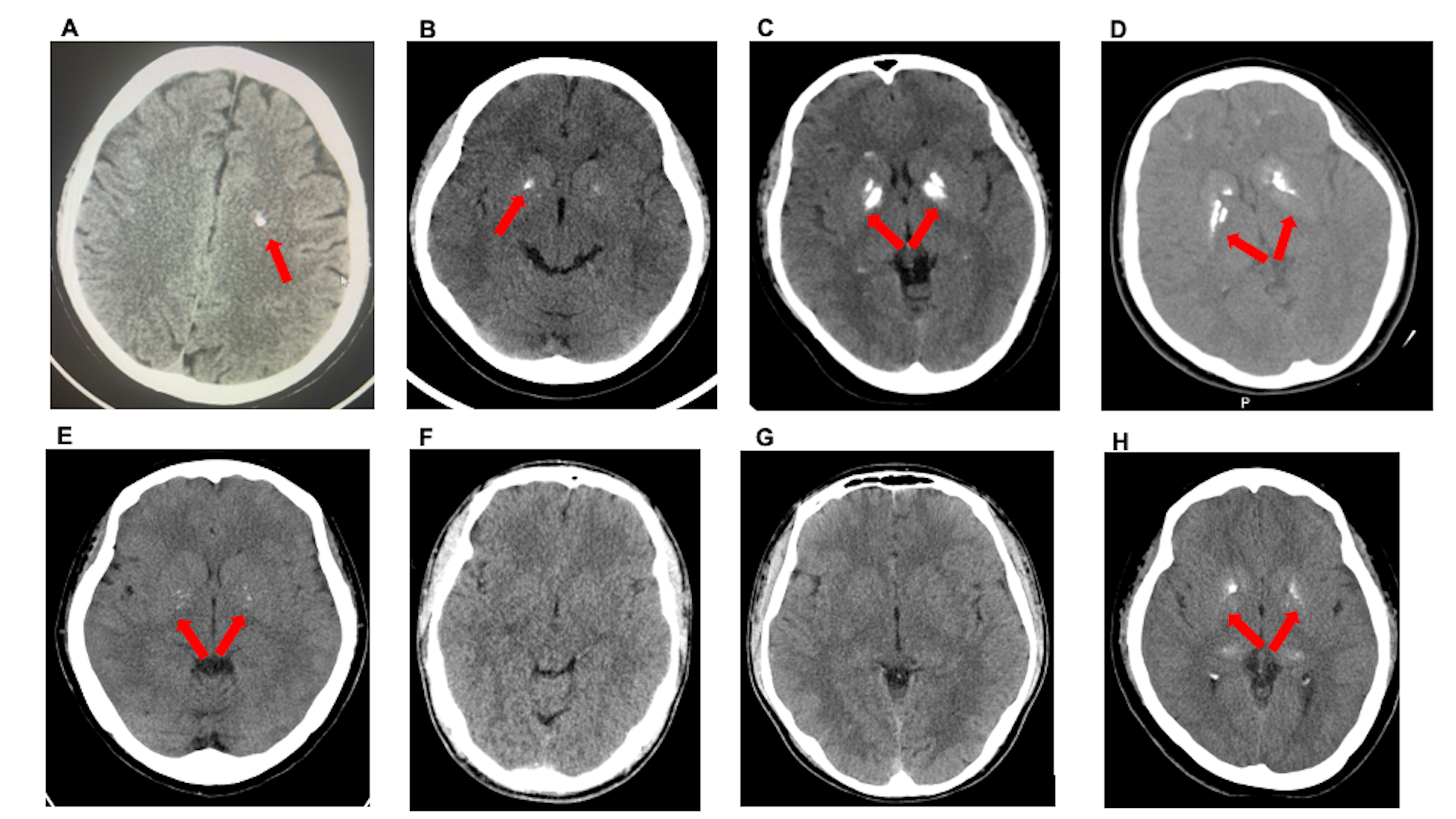

Supplement: Supplementary file 1 [file Image1.tiff]

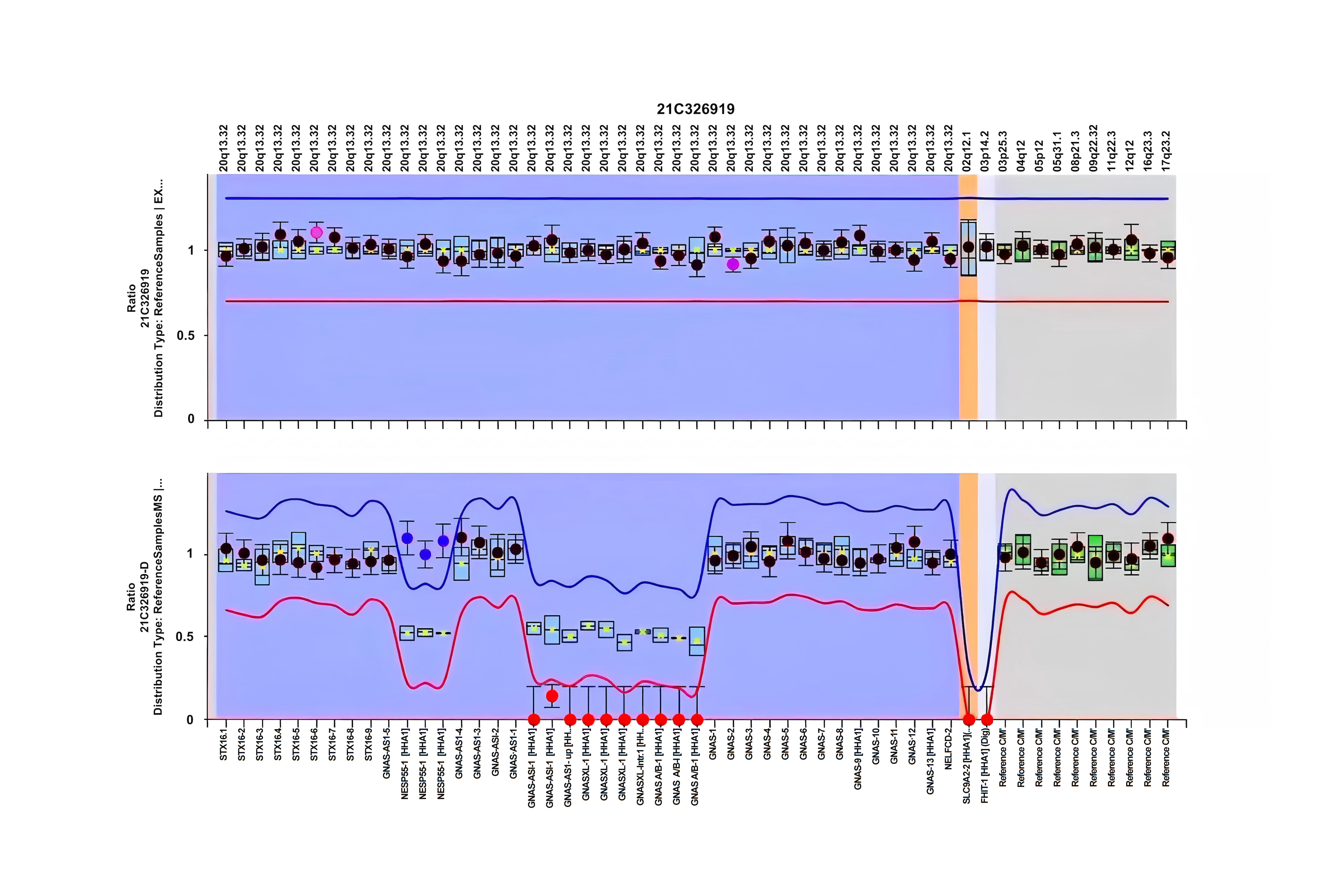

Supplement: Supplementary file 3 [file DataSheet1.zip › Fig1C-case3.tif]

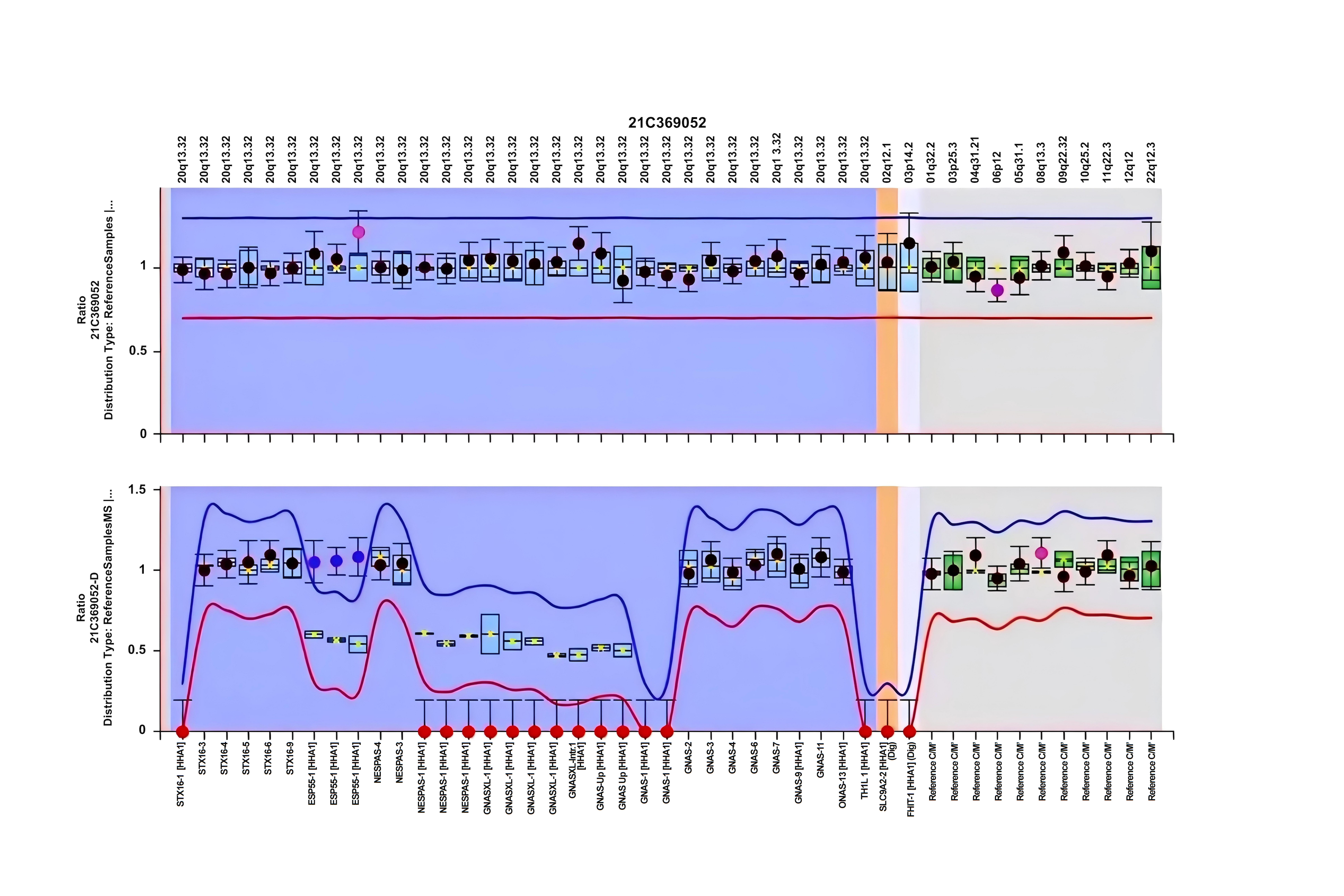

Supplement: Supplementary file 3 [file DataSheet1.zip › Fig1D-case4.tif]

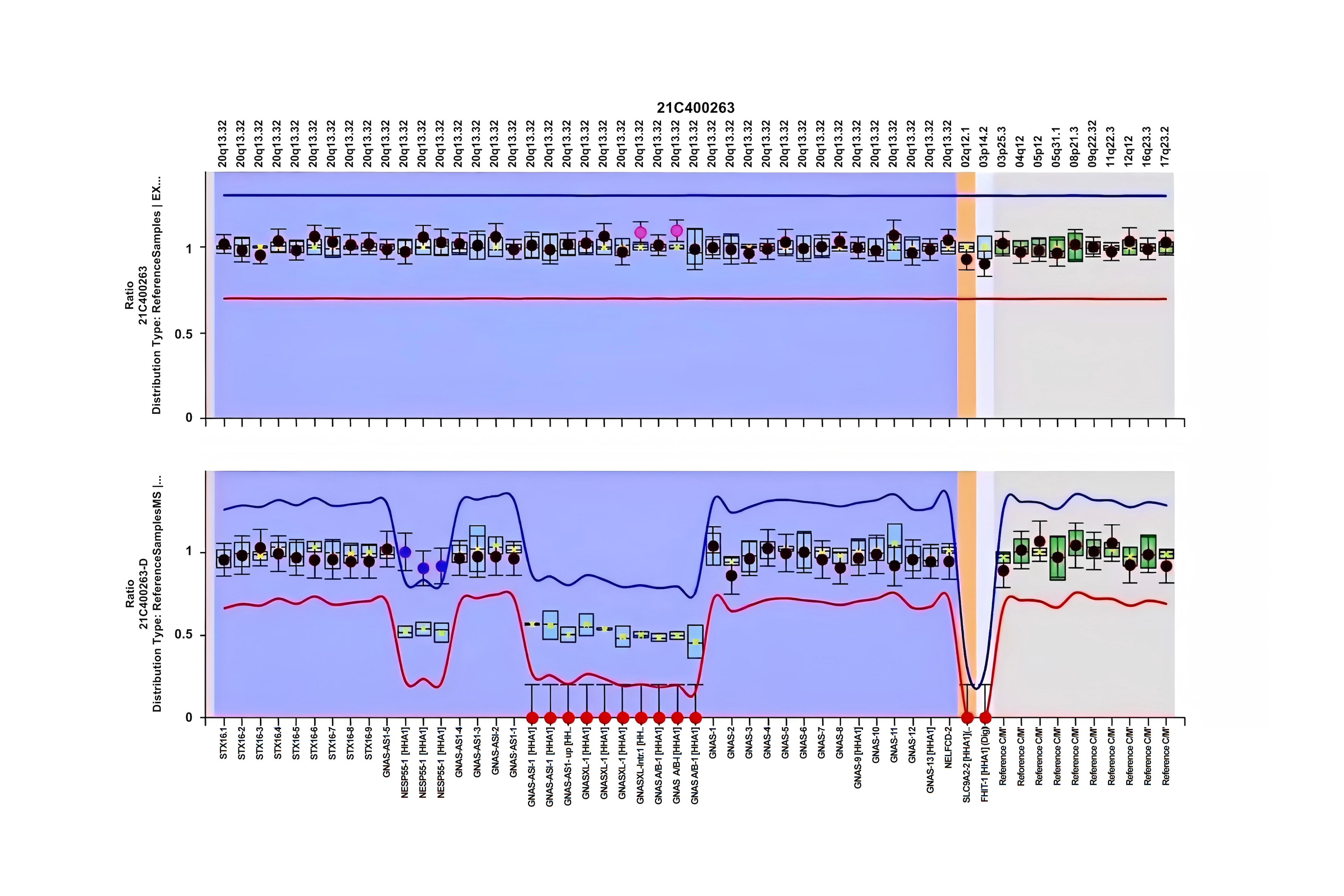

Supplement: Supplementary file 3 [file DataSheet1.zip › Fig1A-case1.tif]

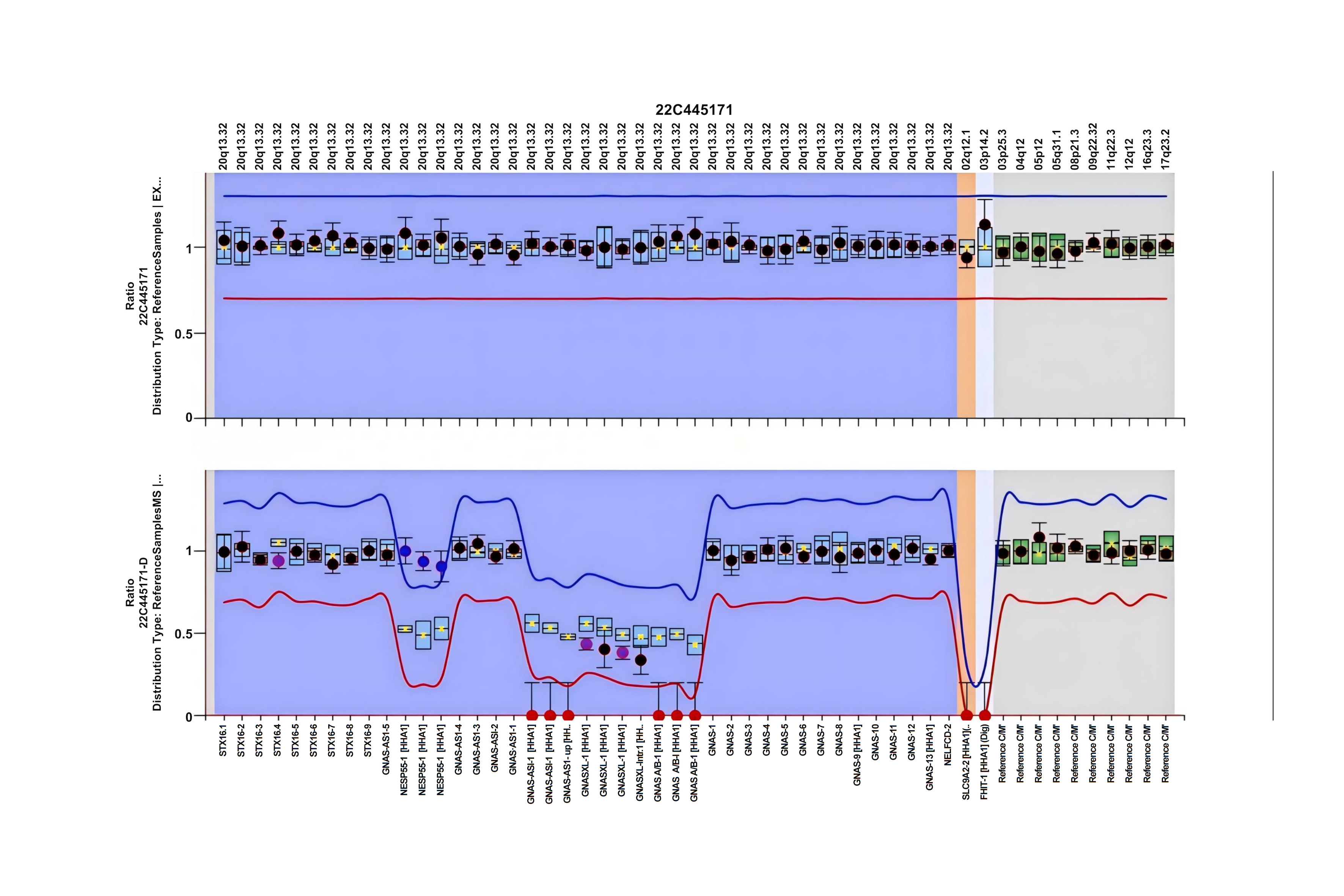

Supplement: Supplementary file 3 [file DataSheet1.zip › Fig1B-case2.tif]

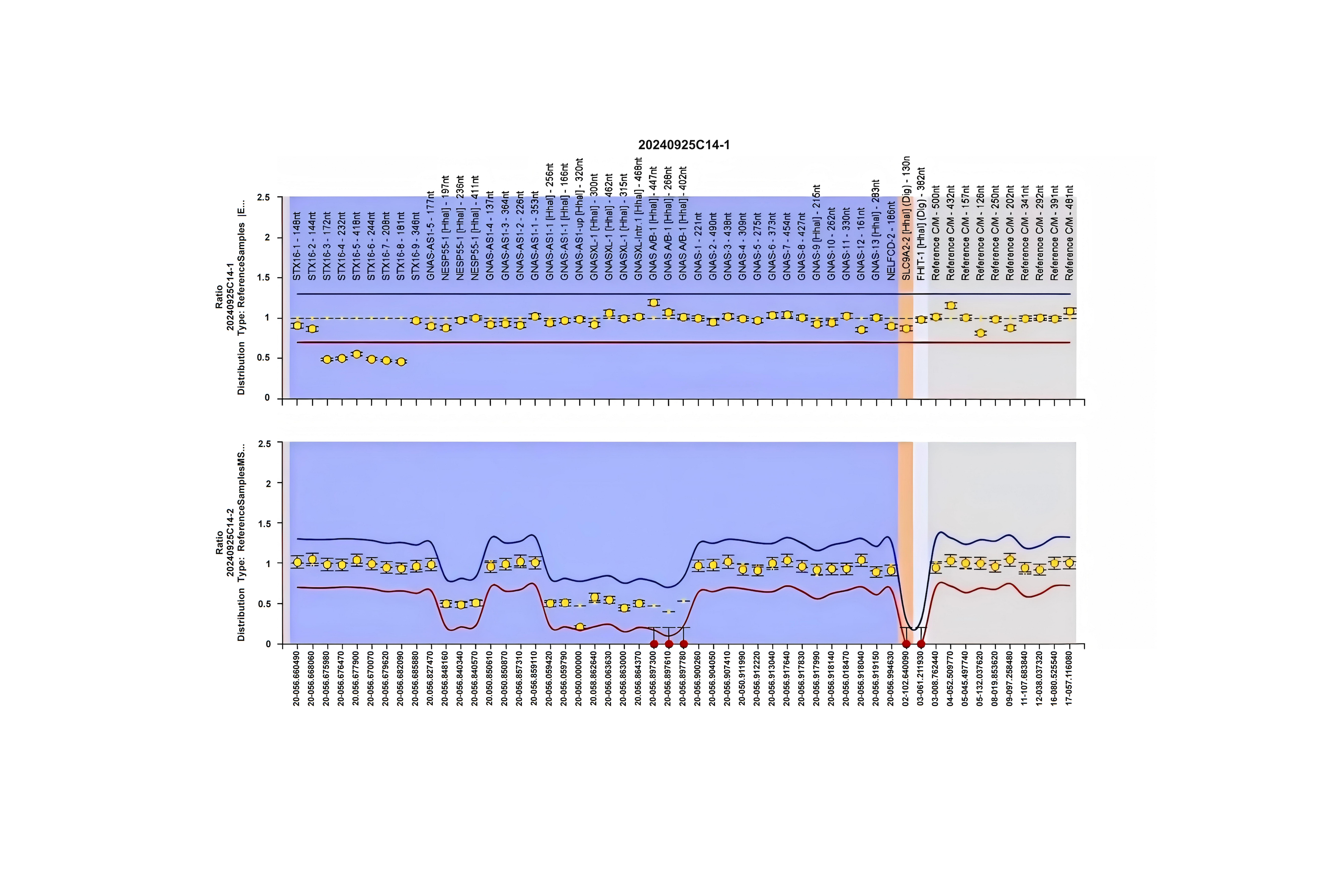

Supplement: Supplementary file 3 [file DataSheet1.zip › Fig1E-case5.tif]

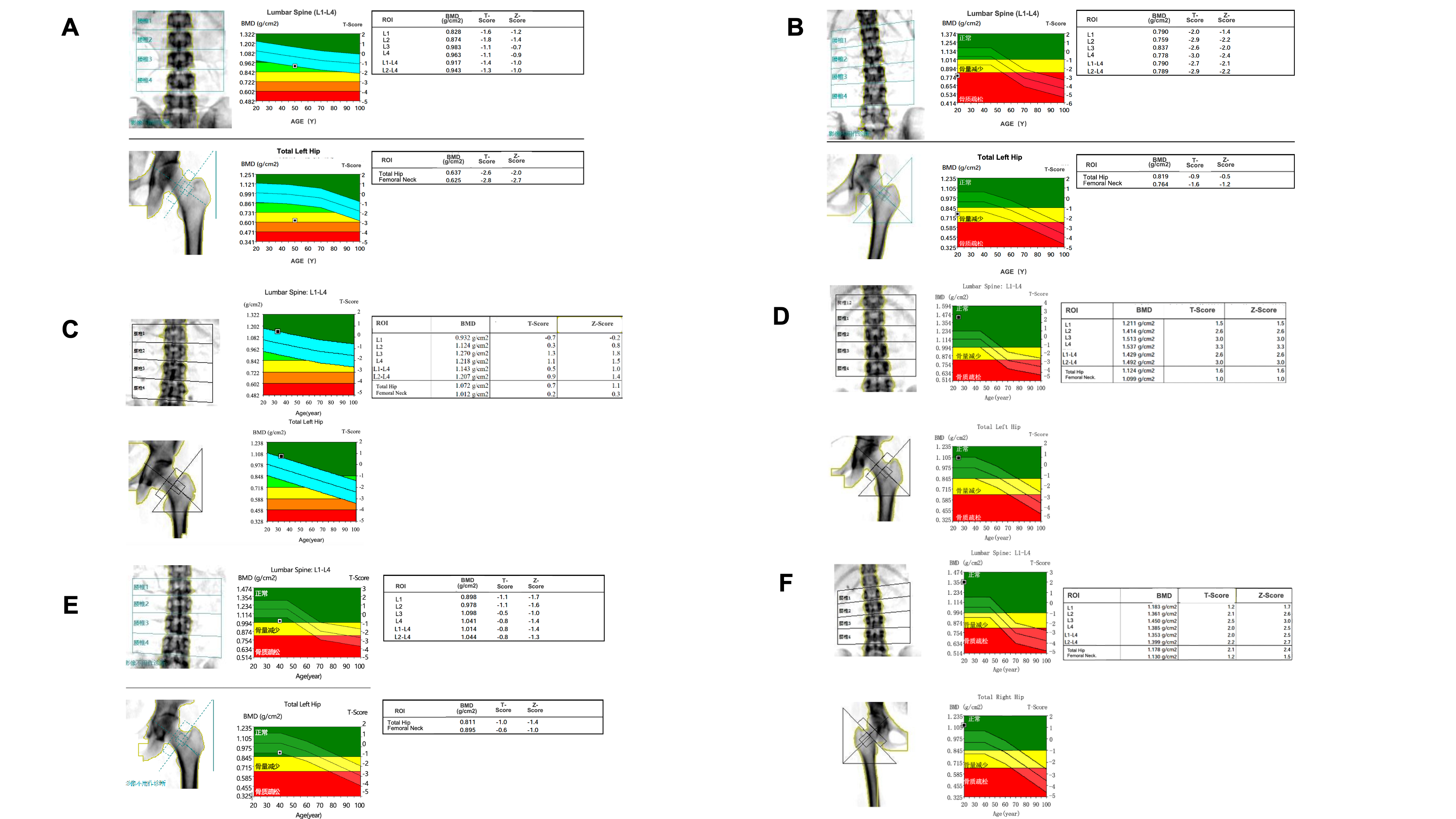

Supplement: Supplementary file 4 [file Image2.tiff]
