# Supplementary material for: Pseudohypoparathyroidism type 1B mimicking gitelman syndrome: diagnostic pitfalls and molecular insights
Source: Front Genet. 2025 Aug 14;16:1638472. doi: 10.3389/fgene.2025.1638472 (PMC12390988; doi:10.3389/fgene.2025.1638472)
Supplement: Supplementary file 2 [file Table1.docx]

**Supplemental materials:**

**Pseudohypoparathyroidism Type 1B Mimicking Gitelman Syndrome: Diagnostic Pitfalls and Molecular Insights**

**Yiming Zhao^1*^, Lijun Mou^2^, Oumayma Akaaboune^3^, Jiudan Zhang^1^**

^1^ Department of Endocrinology and Metabolism, the Second Affiliated Hospital Zhejiang University School of Medicine, Hangzhou, China. ^2^ Department of Nephrology, the Second Affiliated Hospital Zhejiang University School of Medicine, Hangzhou, China. ^3^ Zhejiang University School of Medicine, Hangzhou, China.

*** *Correspondence:***

Yiming Zhao

zhaoyiming@zju.edu.cn

**Table S1**. Key Comparisons of PHP1B Patients with Hypokalemia, PHP1B Patients without Hypokalemia and GS Patients.

|  | PHP1B with Hypokalemia (n=5) | PHP1B Controls (n=5) | GS Controls (n=5) |
| --- | --- | --- | --- |
| PTH (pg/mL) | 422.1 ± 232.0 | 353.2 ± 170.0 | 35.7 ± 27.5 * |
| Potassium  (mmol/L) | 3.14 ± 0.07 | 3.74 ± 0.05 * | 2.71 ± 0.32 * |
| Calcium  (mmol/L) | 1.55 ± 0.21 | 1.79 ± 0.17 | 2.32 ± 0.17 * |
| Phosphorus (mmol/L) | 1.41 ± 0.22 | 1.69 ± 0.30 | 1.10 ± 0.19 |
| Magnesium  (mmol/L) | 0.74 ± 0.10 | 0.80 ± 0.06 | 0.47 ± 0.08 * |
| Intracranial Calcifications | 5/5 (100%) | 3/5 (60%) | Not performed† |
| Osteoporosis | 2/3 (67%) | 0/3 (0%) | Not performed† |

Data expressed as mean ± SD; *P<0.05 vs. PHP1B with hypokalemia; †GS lacked indications for cranial CT or DXA scanning.

PHP1B, Pseudohypoparathyroidism Type 1B; GS, Gitelman Syndrome; PTH, parathyroid hormone.

Reference ranges: PTH, 15-65 pg/mL; potassium, 3.5-5.3 mmol/L; calcium, 2.11-2.52 mmol/L; phosphate, 0.85-1.51 mmol/L; magnesium 0.75-1.02 mmol/L; Osteoporosis, Z-score ≤ -2.0 in premenopausal women and men <50 years, T-score ≤ -2.5 in postmenopausal women and men ≥50 years at lumbar spine/femoral neck.

**Figure Legends**

**Figure S1**. Non-contrast head computed tomography (CT) images revealed calcifications (arrows) in all hypokalemic PHP1B patients (A-D: Cases 1, 3, 4, and 5, respectively), but only in 60% (3/5) of PHP1B patients without hypokalemia (E-H). (Case 2 is shown in the main text Figure 2). Control subjects exhibited the following: (E) Case 1 (39-year-old female) had bilateral basal ganglia calcifications; (F) Case 2 (27-year-old male) showed a normal CT scan; (G) Case 3 (15-year-old male) showed a normal CT scan; (H) Case 4 (27-year-old male) had bilateral basal ganglia calcifications. Images for Control Case 5 (20-year-old female with intracranial calcifications) were unavailable as the scan was performed externally.

**Figure S2**. Bone mineral density (BMD) scans were performed in six PHP1B patients. Among the three patients with hypokalemia scanned, Case 1 (A) and Case 3 (B) demonstrated osteoporosis, while Case 2 (C) showed BMD within the normal range. Scans of three PHP1B patients without hypokalemia revealed normal BMD: Case 4 (27-year-old male, D), Case 1 (39-year-old female, E), and Case 5 (20-year-old female, F).
